# Accuracy of mHealth Devices for Atrial Fibrillation Screening: Systematic Review

**DOI:** 10.2196/13641

**Published:** 2019-06-16

**Authors:** Godwin Denk Giebel, Christian Gissel

**Affiliations:** 1 Health Economics Department of Economics and Business Justus Liebig University Giessen Germany

**Keywords:** mHealth, atrial fibrillation, wearable, app

## Abstract

**Background:**

Mobile health (mHealth) devices can be used for the diagnosis of atrial fibrillation. Early diagnosis allows better treatment and prevention of secondary diseases like stroke. Although there are many different mHealth devices to screen for atrial fibrillation, their accuracy varies due to different technological approaches.

**Objective:**

We aimed to systematically review available studies that assessed the accuracy of mHealth devices in screening for atrial fibrillation. The goal of this review was to provide a comprehensive overview of available technologies, specific characteristics, and accuracy of all relevant studies.

**Methods:**

PubMed and Web of Science databases were searched from January 2014 until January 2019. Our systematic review was performed according to the Preferred Reporting Items for Systematic Review and Meta-Analyses. We restricted the search by year of publication, language, noninvasive methods, and focus on diagnosis of atrial fibrillation. Articles not including information about the accuracy of devices were excluded.

**Results:**

We found 467 relevant studies. After removing duplicates and excluding ineligible records, 22 studies were included. The accuracy of mHealth devices varied among different technologies, their application settings, and study populations. We described and summarized the eligible studies.

**Conclusions:**

Our systematic review identifies different technologies for screening for atrial fibrillation with mHealth devices. A specific technology’s suitability depends on the underlying form of atrial fibrillation to be diagnosed. With the suitable use of mHealth, early diagnosis and treatment of atrial fibrillation are possible. Successful application of mHealth technologies could contribute to significantly reducing the cost of illness of atrial fibrillation.

## Introduction

### Background

Atrial fibrillation is a cardiac arrhythmia appearing in different forms. Globally, 33.5 million people are affected by atrial fibrillation [1]. This disease leads to a significantly increased risk of all-cause mortality, cardiovascular mortality, major cardiovascular events, stroke, ischemic stroke, ischemic heart disease, sudden cardiac death, heart failure, chronic kidney disease, and peripheral arterial disease [2].

Atrial fibrillation can occur in five different forms: first diagnosed, paroxysmal, persistent, long-standing persistent, and permanent. For patients above 65 years of age, opportunistic screening for atrial fibrillation is recommended by pulse taking or using an electrocardiogram (ECG) rhythm strip. The gold standard for atrial fibrillation detection is the 12-lead ECG [3].

Because atrial fibrillation is a serious risk factor for stroke and mortality, its treatment is inevitable for patients. Through medication with oral anticoagulation such as vitamin K antagonists or nonvitamin K antagonists, the risk for stroke and mortality in patients with atrial fibrillation can be markedly reduced [4,5].

In addition to high health risks for patients with atrial fibrillation, the economic burden of the disease is vast. An investigation carried out by Johnson et al estimated an average of €20,403-€26,544 for the cost of illness caused by atrial fibrillation over 3 years in the Danish health care system [6]. For Sweden and Germany, the cost of illness amounts to €7,241 and €5,586 per year, respectively [7]. In this context, secondary diseases like stroke cause the majority of costs. The difference in costs between treated and untreated atrial fibrillation is significant. A stroke survivor with atrial fibrillation receiving oral anticoagulation costs €17,518, and the cost for a stroke survivor with atrial fibrillation not receiving oral anticoagulation is €19,143 [8]. Furthermore, there are several studies confirming the cost-effectiveness of screening for atrial fibrillation [9-12].

One of the main challenges is detection of irregular forms of atrial fibrillation in an accurate way in order to start treatment as soon as possible. Even an ECG taken over a longer period (>24 hours) using a Holter monitor does not always lead to a reliable diagnosis of existing atrial fibrillation. In the case of paroxysmal atrial fibrillation, the occurrence of the disease often cannot be detected within the first 48 hours of ambulatory ECG monitoring [13].

To manage the increasing number of patients with atrial fibrillation and to cope with the consequences of this disease, an early diagnosis is fundamental. In this context, mobile health (mHealth) has often been suggested as a possible solution.

Among the reviews of the use of mHealth for the diagnosis, treatment, and prevalence estimation of arrhythmias [14-26], only one systematic review focused on outpatient cardiac rhythm monitoring in cryptogenic stroke [16]. Therefore, we conducted a systematic review focusing especially on the most recent and relevant noninvasive mHealth devices for the detection of atrial fibrillation. The aim of this article was to provide a systematic overview about the possible and real application of mHealth as well as to show its potentials and limitations by assessing the measurement quality.

### Mobile Health

Smartphones, tablets, and mobile apps are widely used in many parts of the world. With an increasing rate of usage, two-thirds of the population in Europe and North America own at least one mobile device. Hence, there is already a basis for an mHealth approach in the context of atrial fibrillation, and the incremental costs for its use are relatively low.

There are two possible stages for mHealth use in the context of atrial fibrillation. First, the treatment of atrial fibrillation should start even before the occurrence of arrhythmia, in the form of prevention. Obesity, physical inactivity, and hypertension are preventable risk factors [3]. Despite the fact that behavior does not change by purchasing a wearable device or smartphone, these devices can contribute to a healthier and more active lifestyle [15].

Second, when atrial fibrillation has occurred, there are four possibilities to support the diagnosis and treatment: ECG or rhythm monitoring, heart rate monitoring, symptom and environmental annotation, and medication adherence [26].

Diverse propositions exist in the field of medication support. One approach is to support patients through communication of general knowledge about the disease, the mechanism of medication, and medication adherence [27-29]. Other applications provide guidelines and risk scores to support decision making for treatments [30,31].

In this review, the specific focus is on the diagnosis of atrial fibrillation by monitoring the heart rate and detection of arrhythmia by mHealth devices. For this purpose, event monitors or Holter devices are used. Monitoring can be done by either loop recorders or postevent recorders. The former is used over a long period, wherein electrodes are attached to the skin in order to monitor the heart activity when triggered by patients or an embedded algorithm. The patient-activated postevent ECG is not worn continuously, but used regularly or immediately after symptoms have occurred [32]. Nowadays, especially through the development in the field of mHealth and its simple use outside of health care, both approaches can record cardiac activities in an extensive way and thereby support the diagnosis of atrial fibrillation.

Despite the high cost of illness of atrial fibrillation, there are few economic assessments for mHealth solutions [33,34].

## Methods

Our systematic review is performed according to the guidelines for the Preferred Reporting Items for Systematic Reviews and Meta-Analyses [35].

### Article Retrieval

We ran literature searches in PubMed MEDLINE and Web of Science databases in January 2019. Regarding time of publication, we considered the year 2014 as the baseline, because there are some reviews covering previous years [19,24,26]. As search keywords, we used the terms “mHealth,” “telemedicine,” “wearable,” “mobile health,” “app,” and “digital treatment” in combination with the term “atrial fibrillation.”

### Study Selection

Eligible studies had to meet the following predefined criteria: original research, focus on the diagnosis of atrial fibrillation, interventions using mHealth devices, noninvasive, and published in English language. The following were used as exclusion criteria: focus on technical descriptions or algorithms and lack of information about the accuracy of the investigated device.

**Table 1:** Overview of all studies included in the review.

Study and app/device		FDA^a^ approval	Study population	Reference method	Recording duration (mHealth^b^ device)	Sensitivity, %	Specificity, %	PPV^c^, %	NPV^d^, %
**Bonomi et al [36]**									
	CM3 Generation-3, Connected Sensing	No	AF^e^ patients before and after elective electrical cardioversion (n=20)	Actiwave Cardio (single-lead ECG^f^)	60 min	97	100	99	98
	CM3 Generation-3, Connected Sensing	No	Patients prescribed to undergo 24-/48-hour ECG Holter with either paroxysmal or persistent AF (n=40)	12-lead Holter ECG	Duration of the Holter monitoring period	93	100	N/A^g^	N/A
**Brasier et al [37]**									
	Heartbeats app + iPhone 4S	No	In-house patients with presumed AF and matched controls in sinus rhythm (n=592)	Single-lead iECG^h^ (AliveCor)	1 min	89.9	99.1	N/A	N/A
	Heartbeats app + iPhone 4S	No	In-house patients with presumed AF and matched controls in sinus rhythm (n=592)	Single-lead iECG (AliveCor)	5 min	91.5	99.6	N/A	N/A
**Bumgarner et al [38]**									
	AliveCor KardiaBand + Apple Watch + smartphone	Yes	Patients with a diagnosis of AF who presented for scheduled elective cardio version, aged 18-90 years (n=100)	12-lead ECG	N/A	93	84	N/A	N/A
**Chan et al [39]**									
	Cardiio Rhythm + iPhone 4S	No	Patients with either hypertension or diabetes mellitus or aged ≥65 years (n=1013)	12-lead ECG (15 min)	17.1 s	92.9	97.7	53.1	99.8
	AliveCor Heart Monitor	Yes	Patients with either hypertension or diabetes mellitus or aged ≥65 years (n=1013)	12-lead ECG (15 min)	30 s	71.4	99.4	76.9	99.2
**Desteghe [33]**									
	AliveCor KardiaMobile	Yes	Patients at a cardiology ward (n=320)	12-lead ECG (10 sec)	30 s	36.8	96.1	56	91.1
	AliveCor KardiaMobile	Yes	Patients at a geriatric ward (n=125)	6-lead ECG (30 sec)	30 s	72.7	98.1	88.9	94.4
	MyDiagnostick	No	Patients at a cardiology ward (n=320)	12-lead ECG (10 sec)	30 s	60.5	93.3	54.8	94.6
	MyDiagnostick	No	Patients at a geriatric ward (n=125)	6-lead ECG (30 sec)	30 s	81.8	96.1	81.8	96.1
**Eerikäinen et al [40]**									
	CM3 Generation-3, Connected Sensing	No	Patients before and after an electrical cardioversion procedure in the hospital (n=18)	Single-lead (Actiwave Cardio) ECG and 24-hour Holter	2 h	92.3	60.7	N/A	N/A
	CM3 Generation-3, Connected Sensing	No	24-hour measurements in normal everyday conditions (n=16)	Single-lead (Actiwave Cardio) ECG and 24-hour Holter	24 h	71.6	84.9	N/A	N/A
**Fan et al [41]**									
	HUAWEI Mate 9	No	Patients aged ≥18 years excluding patients with ICD^i^ or pacemaker (n=108)	12-lead ECG (3 min)	3 min	94.41	100	100	95.43
	HUAWEI Honor 7x	No	Patients aged ≥18 years excluding patients with ICD or pacemaker (n=108)	12-lead ECG (3 min)	3 min	95.56	99.4	99.23	96.49
	HUAWEI Band 2	No	Patients aged ≥18 years excluding patients with ICD or pacemaker (n=108)	12-lead ECG (3 min)	3 min	95.36	99.7	99.63	96.24
**Gropler et al [42]**									
	AliveCor KardiaMobile	Yes	Patients aged <18 years with standard 12-lead ECG ordered as part of routine visit testing (n=30)	Standard 12-lead ECG	30 s	N/A	87	N/A	N/A
**Haberman et al [43]**									
	AliveCor KardiaMobile (iPhone case or iPad)	Yes	Division I athletes (n=123)	Standard 12-lead ECG	30 s	N/A	99.2	N/A	100
	AliveCor KardiaMobile (iPhone case or iPad)	Yes	Healthy young adults (n=128)	Standard 12-lead ECG	30 s	N/A	100	N/A	100
	AliveCor KardiaMobile (iPhone case or iPad)	Yes	Cardiology clinic patients (n=130)	Standard 12-lead ECG	30 s	94.4	99.1	94.4	99.1
**Hochstadt et al [44]**									
	CardiacSense	In process	Patients aged ≥18 years excluding patients with ICD or pacemaker (n=108)	Simultaneously recorded ECG	30 min	100	N/A	N/A	N/A
**Kang et al [45]^j^**									
	CPstethoscope + Samsung Galaxy S5	No	Selected study participants (n=46)	Cardiologists using an electronic stethoscope	2 min	94	86	88	92
	CPstethoscope + Samsung Galaxy S6	No	Selected study participants (n=46)	Cardiologists using an electronic stethoscope	2 min	94	79	83	92
	CPstethoscope + LG G3	No	Selected study participants (n=46)	Cardiologists using an electronic stethoscope	2 min	81	100	100	82
**Koltowski et al [46]**									
	AliveCor KardiaMobile	Yes	Patients of an academic cardiology care center (n=100)	12-lead ECG	N/A	92.8	100	N/A	N/A
**Koshy et al [47]^k^**									
	FitBit (Blaze) + Apple Watch (Series 1)	No	Patients in sinus rhythm or with arrhythmias, aged ≥18 years from a coronary care unit, an intensive care unit, and an emergency room (n=102)	12-lead ECG	30 min	N/A	N/A	N/A	N/A

**Krivoshei et al [48]**									
	iPhone 4S	No	Patients with AF or patients in sinus rhythm (n=80)	Heart rate monitor chest belt	5 min	95	95	N/A	N/A
**Lahdenoja et al [49]**									
	Different smartphones, mostly Sony Xperia Z-Series	No	Patients with AF and healthy individuals as the control group (n=39)	Previous diagnosed AF	A few minutes (typically less than 5 min)	93.8	100	N/A	N/A
**Lown et al [50]**									
	Polar-H7	No	Individuals from three general practices aged >65 years with and without AF (n=418)	12-lead ECG	N/A	96.34	98.21	N/A	N/A
	AliveCor KardiaMobile	Yes	Individuals from three general practices aged >65 years with and without AF (n=418)	12-lead ECG	N/A	87.8	98.81	N/A	N/A
	Firstbeat Bodyguard 2	No	Individuals from three general practices aged >65 years with and without AF (n=418)	12-lead ECG	N/A	96.34	98.51	N/A	N/A
	WatchBP	Yes	Individuals from three general practices aged >65 years with and without AF	12-lead ECG	N/A	96.34	93.45	N/A	N/A
**Lowres et al [51]**									
	AliveCor KardiaMobile	Yes	Persons aged ≥65 years entering a participating pharmacy (n=1000)	General practitioner review/12-lead ECG	30-60 s	98.5	91.4	N/A	N/A
**Mena et al [52]**									
	Loop recorder ECG sensor device, Classifier, and a smartphone as central unit	No	Older adults (mean age 73.5, SD 11.8 years; n=100)	ECG by expert cardiologist	N/A	100	96.6	N/A	N/A
**Rozen et al [53]**									
	Cardiio Rhythm + iPhone	No	Patients aged >18 years, scheduled for elective cardioversion (n=98)	Standard 12-lead ECG	3 times 20 s before and 3 times 20 s after cardioversion	93.1	90.9	92.2	92
**Selder et al [54]**									
	AliveCor KardiaMobile	Yes	Population participating in the Hartwacht Arrhythmia program (n=233)	ECG interpreting team led by a cardiologist	30 s	92	95	80	98
**Tison et al [55]**									
	Cardiogram application + Apple Watch	N/A^l^	Sedentary participants undergoing cardioversion (n=51)	Standard 12-lead ECG	≥20 min	98	90.2	90.9	97.8
	Cardiogram application + Apple Watch	N/A^l^	Ambulatory participants (n=1617)	Standard 12-lead ECG	≥20 min	67.7	67.6	7.9	98.1


**William [56]**									
	AliveCor KardiaMobile + iPod	Yes/No	Patients aged 35-85 years with a history of paroxysmal or persistent AF (n=52)	12-lead ECG	30 s	96.6	94.1	N/A	N/A

^a^FDA: Food and Drug Administration.

^b^mHealth: mobile health.

^c^PPV: positive predictive value.

^d^NPV: negative predictive value.

^e^AF: atrial fibrillation.

^f^ECG: electrocardiogram.

^g^N/A: not applicable.

^h^iECG: internet-enabled electrocardiography.

^i^ICD: implantable cardioverter defibrillator.

^j^Accuracy of classification of the heart sounds into a correct category. Atrial fibrillation led to significantly fewer interpretable heart sounds. The app needs further improvement to diagnose atrial fibrillation.

^k^No data available on sensitivity, specificity, PPV, and NPV, but there was a significant correlation between device use and ECG in atrial arrhythmias (Apple Watch: *r*_s_=0.83, FitBit: *r*_s_=0.56; both *P*<.01)

^l^No information available about the series used in the study.

### Data Extraction and Analysis

In the first step, we assessed the studies’ eligibility by focusing on the inclusion and exclusion criteria mentioned above. After searching for the reference method used in the study, we searched for sensitivity, specificity, positive predictive value (PPV), and negative predictive value (NPV) in relevant studies as indicators to evaluate the accuracy of the underlying mHealth device. In addition, we extracted characteristics about the study population and the size of the study population as well as recording duration and Food and Drug Administration (FDA) approval (Table 1).

## Results

### Literature Search

**Figure 1:**
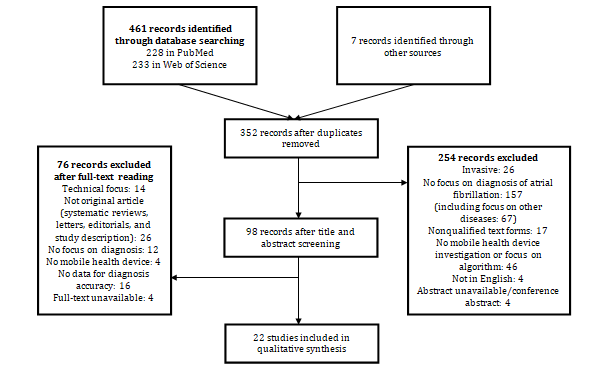
Flowchart of the systematic literature review process.

We identified 461 articles through database searching. We added seven relevant studies either known by the authors, found by searching the reference lists of key studies, or found through a manual search in the Journal of Medical Internet Research and its sister journals. After removing duplicates, there were 352 articles. Of this pool, 22 studies were relevant for our review and included in our work (Figure 1).

To present the results, we categorized the mHealth devices into three groups: apps (“app”), only smartphones or tablets used as a medium for diagnosis, and “wrist worn wearables” and “other devices.”

### Apps

Smartphone or tablet apps are characterized by their usability and the fact that no additional device is needed for atrial fibrillation screening. In this field, a general distinction between direct and indirect photoplethysmography (PPG) can be made. Direct PPGs require direct contact between the user and device. Thus, it is possible to measure the pulse by putting a finger above the camera and flashlight while running the app. Indirect PPGs do not require direct contact; they measure the pulse by scanning a body part over a distance.

Several apps use the direct method. One of the most common smartphone apps in this context is “Cardiio Rhythm,” which can be used either as a direct or an indirect heart rate monitor. Chan et al [39] and Rozen et al [53] investigated the direct use of this app and found a high accuracy in comparison with a single-lead ECG and a 12-lead ECG, respectively.

Krivoshei et al [48] proposed an unnamed app using the direct PPG method. Comparison of the diagnostic results of the app with a heart rate monitor chest belt as a reference method showed high sensitivity and specificity.

Fan et al [41] investigated atrial fibrillation detection through PPG with the aid of either one of two different smartphones or a smart band. Compared to 12-lead ECG, they found high accuracy in both smart phones but concluded that the final diagnosis should be based on ECG. Another study on atrial fibrillation screening with the aid of PPG showed that PPG-based algorithms can reach high accuracy; the authors recommended further investigation using population-based, large-scale atrial fibrillation screening studies [37].

In addition to apps using PPG, there are two fundamentally different approaches. The first one, proposed by Lahdenoja et al [49], is the diagnosis of atrial fibrillation with a smartphone app using the integrated inertial measurement unit. The device is placed on the chest of the patient to measure movement triggered by the heart. Second is the app “CPstethoscope” presented by Kang et al [45] to auscultate the heart. Using this method, they found vast differences in sensitivity, specificity, PPV, and NPV depending on the smartphone model running the app.

### Wrist-Worn Wearables

The most popular wearables used to measure heart frequency and heart rhythm originate from the field of fitness. They appear either as simple bracelets or smartwatches. Besides their potential in supporting basic and clinical research by providing data [57], they have the capability to detect arrhythmias like atrial fibrillation.

In the context of atrial fibrillation, use of such devices can be made in three different ways. First, they can promote healthy behaviors like an active lifestyle. Second, they can support the diagnosis of atrial fibrillation through permanent tracking of heart frequency and rhythm. Lastly, they are able to facilitate coping with the disease [15]. In this review, we focused on diagnosis of atrial fibrillation and the accuracy of mHealth devices.

To detect pulse and heart rhythm, wrist-worn wearables use either PPGs or electrodes. In diagnosis of arrhythmias, the Apple Watch Series 4 is known as the most popular wrist-worn device. It uses both PPG and a two-lead ECG for detection of atrial fibrillation. For the ECG, the first electrode is installed in the digital crown, and the other one is installed on the back of the watch. Thus, this device allows both long-term surveillance of the heart rate through PPG and user-triggered ECG recording with a one-lead ECG.

Koshy et al [47] investigated Apple Watch Series 1 and FitBit Blaze. They showed a high correlation between use of the devices and ECG in patients with sinus rhythm or atrial flutter, but the heart rate in patients with atrial fibrillation tended to be underestimated.

While examining on the Cardiogram app using Apple Watch, Tison et al [55] noted a high accuracy in sedentary patients undergoing cardioversion, but lower accuracy in ambulatory participants.

Another study showed a high accuracy of Apple Watch [38] in combination with the AliveCor KardiaBand, which is evaluated in the AliveCor section below.

In patients with atrial fibrillation, Hochstadt et al [44] found a high correlation between use of such a PPG sensor and simultaneously recorded ECGs in the smartwatch CardiacSense.

Bonomi et al [36] conducted a study on a wrist-worn device that includes a PPG sensor and an accelerometer. When comparing the measurements with either a Holter monitor or a single-lead ECG, the accuracy was high.

Furthermore, while investigating the influence of various conditions on PPGs, Eerikäinen et al [40] found significant differences in sensitivity and specificity between the use in a hospital compared to the use under normal everyday conditions.

Another wrist-worn device, HUAWEI Band 2, was compared to a 12-lead ECG by Fan et al [41]. They found that the PPG smart band is a convenient tool to detect AF at high accuracy.

### Other Devices

Just like wrist-worn wearables, other wearable devices have the capability to measure either the pulse or heart rhythm to detect arrhythmias by using loop or event recording. Most recent articles about atrial fibrillation–diagnosing wearables are either about AliveCor or ECG devices integrated in patches like ZioPatch. Despite its FDA approval, we did not find eligible studies focusing on the accuracy of ZioPatch compared to a reference method. Therefore, it was not part of our review. Due to the high number of studies focusing on the accuracy of AliveCor, we first focused on AliveCor before analyzing studies about other devices.

#### AliveCor

AliveCor KardiaMobile is an event recorder that has been subject to various studies focusing on its accuracy. The device is an FDA-certified medical product that can record heart beat and rhythm by using a single-lead electrode. To measure heart rate and heart rhythm, the user has to put two fingers on the electrodes fixed to a small plastic plate, following which AliveCor KardiaMobile starts to write an ECG and transmits it to either a mobile phone or a tablet computer. It features a very high sensitivity and specificity. Koltowski et al [46] and Lowres et al [51] found high accuracy of this device compared to the standard 12-lead ECG. Furthermore, Selder et al [54] evaluated an arrhythmia program using AliveCor and reported its high accuracy compared to the reference method, which was a team assessing the device-recorded ECGs.

In a study assessing the accuracy of AliveCor in patients with a history of either paroxysmal or persistent atrial fibrillation, William et al [56] found a very high accuracy in the form of sensitivity and specificity.

Nevertheless, analyses with special populations like children [42] and elite athletes and cardiology clinic patients [43], or patients in either a cardiology or geriatric ward [33] showed slightly to significantly modified accuracy compared to the majority of studies focusing on AliveCor.

In a study by Lown et al [50], AliveCore yielded high accuracy but was not superior to inexpensive consumer devices.

A device related to KardiaMobile is KardiaBand. It is a watchband, but its function is similar to that of KardiaMobile; therefore, we deemed evaluation in combination with KardiaMobile appropriate, even though it is a wrist-worn device. A study by Bumgarner et al [38] found very high sensitivity of the KardiaBand used in combination with an Apple Watch (both FDA approved) compared to a 12-lead ECG.

#### Other Devices

Besides the devices mentioned above, there are some, less widespread forms of mHealth for the diagnosis of atrial fibrillation. Few of them have been assessed for accuracy.

MyDiagnostick is similar to AliveCor in its functionality. It is a rod-like device with two electrodes on the endings. Desteghe et al [33] compared the device with either 6-lead or 12-lead ECG for its sensitivity, specificity, PPV, and NPV on patients in a cardiology ward and a geriatric ward. Compared to the algorithm, they found that manual interpretation of the device-recorded data led to increased sensitivity, but decreased specificity.

To detect cardiac abnormalities in the home environment of elderly people residing in low and middle-income countries, Mena et al [52] designed and developed a loop recorder ECG sensor device. Two electrodes are attached to the chest and one to the right leg of the patient. The captured data are directly processed by a machine learning algorithm, and the patient receives feedback through his/her smartphone immediately. Furthermore, the data can be transmitted to health care providers. Tested on 100 older adults, the mobile ECG and the corresponding algorithm reached a very high accuracy (97%), sensitivity (100%), and specificity (96,6%). Thus, further development of the device seems useful.

## Discussion

### Overview

In addition to the devices included in our review, there are many other kinds of mHealth devices to screen for atrial fibrillation, for example, ECG patches like the ZioPatch. Despite its positive evaluation in a multitude of studies [13,58-60], there is no eligible study about its accuracy compared to a reference method. Most of the studies about the ZioPatch compare the detection rate over a given period to the reference method.

To provide an even more accurate diagnosis of atrial fibrillation through mHealth devices, Steijlen et al [61] presented a first approach to allow patients to record an accurate 12-lead ECG at home. They developed a device that can be worn within 8 minutes of first-time use. This device should be studied further.

Another study focusing on the benefit of Apple Watch in the context of irregular heart rhythm detection is the Apple Heart Study [62]. Data about the heart rhythm are received from the Apple Watch and automatically evaluated. If there are irregularities, an app notifies the study participant. Furthermore, there is the possibility for some participants to receive an ePatch and to wear it up to 7 days. After returning the ePatch, the experts offer feedback and recommend further medical care from the participant’s own health care provider. The Apple Heart Study enrolls over 400,000 participants and is thus the largest ever study of its kind. The study results are not yet published.

Despite the overall good evaluation of mHealth devices in the context of atrial fibrillation, there are some possible limitations. Shcherbina et al found that exogeneous factors like dark skin color, higher body mass index, and male gender as well as mechanical separating or shifting of PPG during physical activities led to higher device errors [63]. Furthermore, part of the recorded atrial fibrillation screenings were noninterpretable by algorithms [38,56].

### Principal Results

In this review, we presented various possibilities to screen for atrial fibrillation. mHealth devices appearing in different forms like smartphone apps, wrist-worn devices, small plates such as the AliveCor, or rod-like devices were investigated for their accuracy. These devices mostly use either ECG or PPG technology to detect atrial fibrillation.

Mobile apps provide a convenient way to screen for atrial fibrillation. Most common are apps using PPG, which allows detection of atrial fibrillation with a high accuracy compared to the gold standard. Furthermore, it is possible to develop apps that use the inertial measurement unit or can be used to auscultate the heart.

Wrist-worn wearables appearing as bracelets or smart watches provide the possibility to measure heart rhythm in an unobtrusive way. The most effective way to guarantee atrial fibrillation detection is to combine PPG and ECG in a wrist worn-wearable device in order to screen over a long-term period and record an accurate user-triggered ECG. This is the case for Apple Watch or Apple Watch in combination with KardiaBand.

The ECG-based AliveCor is one of the few FDA-approved devices. It reaches a very high overall accuracy and benefits from its ease of use. Overall, the use of mHealth devices is convenient [34,61,64,65]. Nevertheless, after atrial fibrillation detection through mHealth devices, the diagnosis should always be confirmed by standard 12-lead ECG Holter monitoring.

### Economic Aspects

From an economic point of view, mHealth devices seem to be an eligible possibility to prevent expensive secondary diseases like stroke. Therefore, mobile apps have a high economic potential in screening for atrial fibrillation. Given the fact that smartphones are already widespread in many countries, the economic burden is low. Even if app accuracy does not reach the gold standard, mobile apps can provide a first approach to detect atrial fibrillation.

The integration of atrial fibrillation screening methods in smartwatches and bracelets could be valuable. Smart watches, in particular, have gained popularity during the last few years. Further investigation on the economic effect of subsidizing wrist-worn wearables, which are able to screen for atrial fibrillation, should be performed. A special focus should be placed on the accuracy of these devices to avoid costs due to misdiagnosis.

With a fundamentally different approach, AliveCor benefits from its ease of use. This device seems suitable to integrate in health care as already implemented in the Dutch Hartwacht program [54]. Orchard et al implemented a study to examine the cost-effectiveness of screening with AliveCor in a rural primary care setting. The aim was to screen 2000 patients aged ≥65 years for atrial fibrillation during 3-4 months and to evaluate the process through qualitative interviews as well as cost-effectiveness [56]. Especially for low-income countries, mHealth is a possible approach to screen for atrial fibrillation, which will reduce the economic burden [66,67]. Nevertheless, to assess the real economic potential of mHealth devices in the context of atrial fibrillation screening, further studies for all types of mHealth devices are needed.

### Conclusions

The main advantage of mHealth in atrial fibrillation detection is its use in addition to standard care. Even if its accuracy is not yet as high as expected, it is an additional possibility to diagnose atrial fibrillation, especially in its silent, paroxysmal form. Economic assessment of mHealth devices should be further explored.

## Abbreviations

AF: atrial fibrillation
ECG: electrocardiogram
FDA: Food and Drug Administration
mHealth: mobile health
NPV: negative predictive value
PPG: photoplethysmography
PPV: positive predictive value
